# Body image in paediatric burns: a review

**DOI:** 10.1186/s41038-018-0114-3

**Published:** 2018-04-29

**Authors:** Ian C. C. King

**Affiliations:** Department of Plastic and Reconstructive Surgery, St George’s Hospital, Tooting, London, SW17 0QT UK

**Keywords:** Burns, Body image, Children, Paediatric burns, Wound healing

## Abstract

Burn injuries in children can result in life-long disfigurement. As medical and surgical techniques of burn management improve survival prospects more than ever before, body image adjustment is increasingly a central consideration in the care of burn-injured individuals. An appreciation that both physiological and psychosocial processes underpin such injuries is key to understanding wound healing. Perceptions of idealized body images in Western society challenge children and their families as they grow up with and adapt to disfigurement from burns. Whilst many studies have examined the psychosocial recovery of adults with burn injuries, few have considered the impact on burn-injured children. This paper explores the models of body image and discusses the relevance of these to research and practice in understanding how to manage burns in children.

## Background

Burn injuries can result in life-long disfigurement for children. With advances in medical and surgical techniques of resuscitation, healing and reconstruction, people sustaining burn injuries have better survival prospects than ever before [1, 2]. Body image adjustment is increasingly being recognized as a central consideration in the care of individuals living with burn injuries [3]. The understanding of wound healing requires a holistic appreciation of both physiological and psychological processes initiated at the point of injury [4]. The preoccupation of Western society and media with the notion of the ideal body—attractive, young, slim and blemish-free [5]—is ubiquitous and challenging to children and their families growing up with and adapting to disfigurement from burns. Coping with burn injuries and changes to body image relies on complex interactions of dynamic psychosocial and individual factors which evolve and adapt with time [6]. Whilst many studies have examined the psychosocial recovery of adults with burn injuries, few have considered the recovery of paediatric burn patients. This paper explores the models of body image and discusses the relevance of these to research and practice in understanding how to manage burns in children.

## Review

### Body image

Definitions of body image have evolved since Head, in 1920, first described the concept as a unity of past experiences organized in the cerebral sensory cortex [7]. Indeed, early concepts of body image were rooted in neuropathology, such as the belief that brain damage resulted in a distorted perception of the self [8]. Schilder, a neurologist, introduced a biophysical approach to body image, defining it as the picture we form of our body in our mind, combining psychological attitudes with physical and sociocultural perceptions [9]. Newell observed that body image was dynamic, changing with age, mood or even clothing [10]. Krueger elaborated, suggesting body image is the representation of identity derived from collective internal and external body experiences [11].

### The body image care model

The view of body image being, “the combination of how an individual feels and thinks of their own body and its appearance” is widely understood [12]. Price’s body image care model (BICM) comprises three related elements: body reality, body presentation, and body ideal. *Body reality* is the objective form or phenotype of the body, the result of genetic and environmental influences. *Body presentation* refers to how the body is presented externally, through dress, alteration and behaviour. *Body ideal* is how an individual would like to appear and behave both physically and functionally [12].

The balance of these three elements is crucial to the sustenance of what Price calls a satisfactory body image, whereby both body presentation and body reality are continually, consciously or subconsciously compared with body ideal [12]. The nature of this model’s elements, fluctuating with personality, culture and time suggests body image is dynamic [13]. Tagkalakis and Demiri support this: as individuals change their appearance—either in reality or presentation—body image does not necessarily change; how such changes are interpreted or negotiated against the body ideal is key to maintaining the balance [14]. Altered body image depends on adaptability of all components based on personal experience and expectation.

Price’s model however does not provide a clear definition of what a *satisfactory* baseline body image is from which to measure positive or negative change [10, 14]. If body presentation, reality and ideal were all low, balance would be attained, but a body image would not be satisfactory [10]. Further, many of the assumptions behind the model comprising interacting elements have not been tested empirically [15], drawn instead from subjective clinical observations. Indeed, there is no evidence beyond anecdote to support the existence, let alone the interaction, of these elements [13]. Nonetheless, this model provides a useful framework for healthcare professionals considering body image.

### The fear-avoidance model

Newell’s fear-avoidance model (FAM), based on cognitive-behavioural work on body dysmorphic disorders, attempts to explain why people with disfigurements may or may not reintegrate well into society [10]. This theoretical model developed from the FAM of exaggerated pain perception which regarded fear as having two extreme responses: confrontation and avoidance [16]. It argues that five individual and environmental elements of a patient’s life—namely life events, personality, history of changes to body image, body-image coping strategies, and fear of the changed body and reactions of others to such change—combine to create and influence a psychosocial context in which avoidance or confrontation occurs [10]. These cumulative elements logically develop with age; very young burn-injured children may have little by way of experience to draw upon prior to injury by comparison to children in their teenage years.

Newell theorised that those confronting their anxieties had a better psychosocial recovery than those people who avoided them. Fear of anxiety rather than fear itself determined whether certain behaviours were exhibited [10] and social reinforcement could develop into avoidance of such behaviours with time. Partridge argues that this can prevent the development of adequate coping strategies [17]. As with other models, however, Newell acknowledges the FAM to be speculative, and caution in applying this model to patients with acquired disfigurements is advised [10, 18]. Whilst body dysmorphic disorder demonstrates exaggerated preoccupations with perceived flaws in bodily appearance [19], burn-injured people may nonetheless employ similar avoidance tactics and share the same fears of the reactions of others [10].

### Body image development in children

Primary socialisation begins early in childhood, and a sense of recognition of self is said to develop by the age of two [20]. Once aware of their body appearance, children manipulate parents to receive praise and acceptance [21]. This need for approval widens upon starting school, developing a need for social acceptance [21]. Cash accordingly postulates that body image is a learned behaviour [22]. Smolak suggests that pre-school children largely focus on appearance in the context of the toys they use [20]. Playing with Barbie dolls, hair and clothing instils cultural values and introduces perceptions of body ideal and presentation. The desire of little children to be bigger indicates that as children grow and socialize, they develop comparisons with other children, particularly concerning appearance [20]. Shape, particularly muscle and weight become increasingly prominent considerations by the age of 6 [20]. Indeed, Smolak reported that 40–50% of junior school children aged 6–12 years old demonstrated dissatisfaction with some element of their body size or shape [20]. Adolescence marks the transition from childhood to adulthood and carries with it associated physical and social changes [23]. Factors such as gender, fashion, peer group relations, educational and familial influences and evolving socialization blend with physical changes such as hair growth, acne, breast development and menstruation to situate even non-burned children into unfamiliar territory with vulnerable body images.

The majority of research concerning body image in children focuses on weight and shape concerns. Accordingly, most models of body image in children are rooted in the research of eating disorders [24] with a focus on body image in girls, rather than boys. Cusamano and Thompson found 40-70% of uninjured adolescent girls to be unhappy with at least two aspects of their bodies, with 50–80% reporting that they would like to be thinner [25]. The phrase ‘normative discontentment’ was applied, though no results were reported for adolescent boys despite dissatisfaction noted in weight and shape by this group [25]. Indeed, boys are largely overlooked with respect to body image: in 2001, only 17 papers were found to have looked at body image in males younger than 18 years old [26].

### Body image in children with burns

Pope et al. compared burn-injured and non-burned adolescents through questionnaires assessing mood, body image and quality of life (QOL) [27]. A mixed comprehensive school served as a control, and children with burns were recruited through their parents based upon records of admission to a regional burns unit or attendance at burns camps. A total of 36 burn survivors replied (13 boys, 23 girls), as did 41 school control children (18 boys, 23 girls). Mean ages were identical for each group: 15.1 years old (ranges of 11–19 and 12–19 respectively). Burns occurred on average 11 years 9 months before the study and had a mean size of 22.5% total body surface area (TBSA, range 1–63%). The results identified significant differences between genders in both burn-injured and control groups concerning feelings about appearance; boys were generally more positive (*p* = 0.001). Riccardelli and McCabe hypothesised that boys often focus on the positive aspects of their bodies as protective and adaptive responses to change [28].

In keeping with adult studies [29], Pope et al. also found that female burn-injured adolescents expressed more negative evaluations of how others saw their appearance than burn-injured males (*p* = 0.012), but overall, burn-injured adolescents reported more positive—if not statistically significant—feelings about their appearance than the controls [27]. Brown et al. however found no difference between the sexes in terms of psychosocial adjustment [30].

Significantly, the burn-injured population in the Pope et al.'s study also expressed more positive evaluations of how others see their appearance than the control groups (*p* = 0.018) and were less concerned by their weight (*p* = 0.001). Overall, the burn-injured respondents reported a higher QOL than the controls (*p* = 0.005) [27]. In applying Price’s BICM, it may be that the body ideal differs importantly between the sexes to bring about body image differences, but more likely is that confrontation of the challenges in body image over an average nearly 12 years, as described by Newell, shapes a more secure idea of body image in burn-injured children in comparison to their non-burned peers. Care of course must be taken in interpretation of such results; the questionnaires were sent to parents which may have influenced which children returned the questionnaires (and how they were filled out) and the 36.7% response rate may represent a response bias. Non-responders did not appear to be followed-up in Pope et al.’s study. No two studies appear to use the same questionnaires, and research methodology varies widely, making comparisons of results difficult. Furthermore, 75% of burn-injured children attended burns camps which support children in addressing body image concerns, and therefore, such results may not be representative of all burn-injured adolescents.

### Multiple operations

Burn injuries may require multiple surgical interventions. Price’s BICM suggests that for alterations to be accepted, the individual must have a clear and realistic set of expectations of the outcome of the operation(s) [12]. A recent study by McGarry et al. included 12 burn-injured children who had required operations (for 1–20% TBSA burns) [31]. Using a phenomenological approach, the authors explored the children’s experiences. With an equal gender ratio, unstructured interviews at 6 months post-burn with children aged 8–15 years old showed that avoidance was common and expectations unrealistic. Photographs were found to be helpful as they showed progress of healing to children. Price proposed that discrepancies in expectations illustrate mismatches between body reality and body presentation in young children [12]. The use of a phenomenological approach in McGarry et al.’s study however potentially limits its transferability to other patients. Phenomenology is deeply rooted in personality and culture, restricted by language used and expression and most importantly by translation and interpretation. Unstructured interviews with a small number of children from a range of ethnicities at one specific time-point in recovery can provide insight into the experience of recovery, but findings are very specific to the studied population, as is typical of qualitative research of this type.

### Adjustments over time

Nonetheless, the theme of managing expectations is important in body image development. Since burn healing is a process occurring over time, the therapeutic relationship between the individual and the therapeutic team is significant in redefining the individual’s body reality [32]. Some research has suggested that positive adjustment to disfigurement occurs naturally over time [33]. Thombs et al. found that people with acquired disfigurement go through an initial development period in which body image worsens but with time, once the social skills needed to cope with their experienced stigmatization develops, it improves again [34]. Research into disfiguring conditions suggests that the severity of the disfigurement does not predict distress [35]; rather, it is the individual’s perception of the disfigurement which is important [36]. Pope et al. however found that injury and perception correlated in their adolescent study [27].

Perceptions of disfiguring injuries appear to change with time. Stubbs et al. considered the impact of facial burns on the psychosocial adjustment of both children and parents in the first 2 years post-injury [37]. Three hundred ninety children aged 0–18 (average 7.3 years old) who sustained burns in a critical area, i.e. hands, genitalia, or burns greater than 20% TBSA (mean 35.5%) were followed up for 24 months after treatment via questionnaires. Psychosocial improvement reported by parents and children of all ages coincided with scar maturation and the time at which pressure garments and active scar prevention were stopped [37]. Patients had largely accepted that the scar was as good as it would ever be; pressure garments and scar prevention might be perceived as a method of confrontation as per Newell, a component of care empowering the patient to influence their supposed body reality and presentation [10]. Parents and children universally were most challenged by facial grafting which took the most getting used to; again, such disfigurement is harder than burns elsewhere to adapt to as effective treatment of the physical scars requires an element of confrontation [37]. Although 61.9% of participants responded to the study, follow-up timing varied between patients and different standards were applied to data for the under 5s, meaning the results should be considered carefully as they may not truly represent responses from all age of children.

### The influence of family

From the beginning of childhood, family is a prominent influence [24], as children develop needing parental approval [21]. It appears that paediatric burns affect the well-being of both children and their families [38]. An investigation by Browne et al. found that poor adjustment in children with acute burn injuries was related significantly to poorer methods of coping and psychosocial adjustment in the mothers [39]. Using questionnaire-based interviews with mothers of 145 burn-injured children selected over a 12-year retrospective period, and using behaviour scores completed by parents to record their child’s behavioural state, Browne et al. suggested that 15% of children with burns were psychosocially maladjusted and found that poor child psychosocial adjustment correlated with mothers demonstrating avoidance behaviours, in keeping with Newell’s FAM [10, 39]. It may be worth considering the extent to which the body ideal held by a parent for their child is transferred to the child itself and what role this plays in the formation of the child’s own body image. That evaluations of child behaviours were conducted by such potentially anxious parents should convey caution to those interpreting these findings however as objectivity is potentially impaired; parents not coping are likely to reflect this through their assessments of their child. Indeed, Wright and Fulwiler noted the importance of assessing the child’s viewpoint; since they suggested that mothers of burned children are often emotionally affected, their subjective ratings of their child may be biased and less valid when considering responses to questionnaires [40].

A prospective longitudinal study by Beard et al. further investigated the importance of parental support [41]. Six school-aged children with acute burns were followed over 5 years to assess their adaptation to their injuries. The parents’ role was found to be a fundamental factor in the development of positive adaptation to a change in body image, with children with ‘facilitating’ parents improving more quickly than those without in the acquisition of a positive and developmentally appropriate body image [41]. With only six patients however, and the use of subjective measures of development, caution in the broader application of this study should be used. This does link to Newell’s model however which suggested that the development of skills through social interaction is key to confronting altered body image [10].

Griffiths et al., in a similar way to Bevans et al., argue that by the age of 8, children are considered to have the skills required to report complex concepts such as their own thoughts and feelings [42, 43]. Ryan et al. however argue that it is limiting to restrict the assessment of paediatric burn outcomes to responses from children themselves, and for outcome questionnaires, responses from their parents/guardians should be included [44].

Patient-reported outcome measures (PROM) in burn care are developing well, though in the field of body image, there remains little that addresses the topic directly. The Brisbane Burns Scar Impact Profile assesses health-related QOL in those with burn scars in different age ranges—adults, children aged 8–18 years, carers of children younger than 8 years and carers of children aged 8 years plus [45]. Whilst this tool comes closest to asking about perceptions of body image in children, it does not do so comprehensively. Scores of scar quality and characteristics attempt to demonstrate impact in a numerical manner, and further interview questions focus on the impact of those characteristics, such as itch and pain. Adult questions address sexual function and relationships, but such questioning was not appropriate for the paediatric population. Emotion was a category in relation to how patients of all ages felt they would cope with scars and accept the way that they looked, but as discussed elsewhere, such expressions are limited by the language abilities of the child, both expressive and receptive.

A body of work undertaken by the American Burn Association and the Shriners Hospital for Children over two decades has developed a program in outcome research which has recruited a cohort of 1140 children with burn injuries across four major burn centres in the USA and followed them up over 4 years [46–52]. The Burns Outcomes Questionnaire (BOQ) comprises of a range of tools to assess QOL for burn survivors with an average burn of 33% TBSA (range 0.3 to 99%) and is intended to be a holistic survey including domains focusing on family functioning, behaviour and motor function. It is a well-established tool with proven reliability and validity [53]; however, the domains are not expressly specific for inward and outward behaviours; instead, they provide an indication of wellbeing and functional status, with appearance appearing only as one sub-domain, along with satisfaction with current status and emotional health. Body image is not expressly addressed.

Meyer et al. compared the BOQ scores of burn-injured adolescents (11–18 years old) and their parents and found these scores largely to correlate, with the exception of a number of domains, which included appearance [49]. It was noted that the adolescents’ assessment of their appearance was better than that of the parent (*p* < 0.001). Whether parental anxiety, guilt or lack of understanding contributes to this was not explored, though it has been noted elsewhere that outward behaviours are best assessed by parents but ratings of inward feelings are best assessed by the adolescent themselves [54–56].

### The influence of peers

As children get older, peer support becomes increasingly important. Whilst it has been suggested that for younger children, families hold a greater influence on body image development than friends [57]; Orr et al. found that young people aged 14–27 were strongly influenced by their peers [58]. Focused on young people injured by burns over the previous decade with an average age of 12.7 years old, questionnaires demonstrated that those who perceived that they had more social support, particularly from friends, exhibited greater self-esteem, less depression and more positive body image compared with those lacking peer support. The limitations with this study however lie mainly in its methodology; with only 48% of 250 patients responding, the results may be a consequence of selection bias. Burn-injured patients with low body image exhibiting avoidance strategies of coping may not have replied, as might those who were unconcerned with their burns. The sex distribution of patients is unstated, as were the extent or locations of the burns and the sort and extent of psychological support required by and offered to these patients following their injuries, information helpful to understand impact and coping strategies considered by different patients. Care should be taken in applying these conclusions, yet they add to a breath of understanding of how body image can be influenced by peers.

### Negotiation

For burn-injured children, it appears that negotiation of body image is no simple feat. The challenges of developing must be compounded by burn injuries which change the body reality that makes, for some, a particular body ideal impossible. As children grow, burn injuries evolve and mature; scar contractures may be unsightly, painful and functionally limiting, requiring further surgical intervention or adaptation which changes the course of body image negotiation entirely. The response to this constantly changing body reality is vital to outcomes for people with burn injuries. An individual’s body image changes constantly and unpredictably throughout their life depending on their cumulative social and personal experiences and perceptions [59].

## Conclusion

The models and research discussed here provide insight into the multifactorial elements influencing children living with burn injuries. The influence of the trio of body reality, presentation and ideal, integrating psychosocial features with biological changes to achieve supposed normality, is constantly in a state of flux. Advances in pharmacology and surgery can help address physical changes and buffer the disparity between body reality and ideal, but psychosocial support is essential to address elements which bring about distress as a consequence of disfiguration and nurture social re-integration in a patient-centered manner. Evaluation of psychosocial interventions is needed with a view to improving the outlook for children who will have life-long burn injuries. Body image in children with burns is dynamic and individual but should not necessarily be addressed alone.

## Abbreviations

BICM: Body image care model
BOQ: Burns Outcomes Questionnaire
FAM: Fear avoidance model
PROM: Patient-reported outcome measure
QOL: Quality of life
TBSA: Total body surface area
